# The Impact of Data on Structure-Based Binding Affinity Predictions Using Deep Neural Networks

**DOI:** 10.3390/ijms242216120

**Published:** 2023-11-09

**Authors:** Pierre-Yves Libouban, Samia Aci-Sèche, Jose Carlos Gómez-Tamayo, Gary Tresadern, Pascal Bonnet

**Affiliations:** 1 Institute of Organic and Analytical Chemistry (ICOA), UMR7311, Université d’Orléans, CNRS, Pôle de Chimie rue de Chartres, 45067 Orléans, CEDEX 2, France; pierre-yves.libouban@univ-orleans.fr (P.-Y.L.); samia.aci-seche@univ-orleans.fr (S.A.-S.); 2 Computational Chemistry, Janssen Research & Development, Janssen Pharmaceutica N. V., B-2340 Beerse, Belgium; jgomezta@its.jnj.com (J.C.G.-T.); gtresade@its.jnj.com (G.T.)

**Keywords:** protein–ligand, binding affinities, deep learning

## Abstract

Artificial intelligence (AI) has gained significant traction in the field of drug discovery, with deep learning (DL) algorithms playing a crucial role in predicting protein–ligand binding affinities. Despite advancements in neural network architectures, system representation, and training techniques, the performance of DL affinity prediction has reached a plateau, prompting the question of whether it is truly solved or if the current performance is overly optimistic and reliant on biased, easily predictable data. Like other DL-related problems, this issue seems to stem from the training and test sets used when building the models. In this work, we investigate the impact of several parameters related to the input data on the performance of neural network affinity prediction models. Notably, we identify the size of the binding pocket as a critical factor influencing the performance of our statistical models; furthermore, it is more important to train a model with as much data as possible than to restrict the training to only high-quality datasets. Finally, we also confirm the bias in the typically used current test sets. Therefore, several types of evaluation and benchmarking are required to understand models’ decision-making processes and accurately compare the performance of models.

## 1. Introduction

The importance of in silico work in the drug discovery pipeline has been growing for several decades. Since the 1980s, numerous drugs have been successfully marketed after being initially designed with the help of computers [1]. Approaches for computer-aided drug design, aiming to identify lead compounds, have steadily improved over time. In structure-based drug design (SBDD), docking is a method that predicts the mode of binding of a molecule into a pocket protein and the affinity of such molecules for the protein target using a scoring function. This method helps in identifying molecular hits in drug design projects. A cornerstone step in this process is to evaluate accurately the binding affinity of the protein–ligand complexes. To this end, various scoring functions, such as knowledge-based, empirical, and force field-based methods, have been developed [2]. The development of scoring functions has advanced further with the integration of machine learning models for bioactivity assessment. Recently, neural networks have gained attention for predicting the binding affinity of protein–ligand complexes. With the advent of big data and access to increased computing power, DL algorithms have emerged as promising tools for prediction purposes. These algorithms harness the structural information of protein–ligand complexes to predict binding affinities, often outperforming other scoring functions [3]. There are also alternative methods for calculating absolute binding free energies, including MMGB(PB)SA [4] and LIE [5]. Additionally, TI and FEP [6] can provide highly accurate predictions, typically within one order of magnitude in affinity, although it is primarily used for relative binding free energy calculations. However, these methods rely on computationally expensive molecular dynamics simulations. Therefore, in virtual screening scenarios, less computationally intensive approaches like deep learning (DL) models are favored. Nevertheless, despite the implementation of new deep neural networks, the performance of the statistical models is stagnating [7].

The performance with DL algorithms relies heavily on the amount of data available to train the statistical models. Unfortunately, the amount of data available for the prediction of binding affinity is relatively low in comparison to other application domains where DL has been successfully applied, like computer vision [8]. Indeed, for binding affinity predictions, models can be trained with the 3D structure of protein–ligand complexes, which are determined by crystallography, NMR, or cryogenic electron microscopy (cryo-EM). On top of this, it is required to perform biophysical experiments, like surface plasmon resonance (SPR), isothermal titration calorimetry (ITC), or more common biochemical assays, in order to evaluate the binding affinity of the complexes. All of these experiments require extensive work, therefore complicating the generation of new reliable data in this field.

We decided to evaluate the different variables related to the data to assess their impact on performance. First of all, a crucial question is to evaluate the minimum amount of data necessary to achieve satisfactory performance. Would 10,000 complexes be enough, or are at least 100,000 required, etc.? To add to these considerations, it is important to keep in mind that an increase in data complexity leads to higher data size requirements. This is especially true for 3D structural data, which are of higher complexity in comparison to most usual deep learning applications. The current state-of-the-art structural-based affinity prediction models are typically trained on the PDBbind [9] dataset. This dataset comprises 3D structures of protein–ligand complexes with known binding affinity (K_D_, K_i_, or IC_50_). In the case that several forms of binding data were available for a complex, K_D_ was selected over K_i_, and K_i_ was selected over IC_50_. This dataset contains 19,443 complexes in its current version (v.2020). Despite the size of the PDBbind increasing every year, having more data is not translated into better performance for the underlying models [7]. One of the main reasons is that the data lacks large series of molecules targeting the same protein, as well as having the same molecule in complex with several proteins. It is proposed that the sparsity of the protein–ligand matrix makes it harder for DL to learn from interactions. On top of this, some teams decided to focus on training on complexes of better quality instead of training on all the data available. In order to validate this approach, we analyzed previously reported models trained on the whole PDBbind and solely PDBbind’s high-quality subset, known as the refined set. Furthermore, we have trained several models with Pafnucy [10], a well-known CNN for the prediction of binding affinities, on both datasets.

Protein–ligand complexes are dynamic, and the binding free energy as ligand passes from solvent to protein represents the energy difference between the ensemble of bound and solvated states. To accurately predict the binding affinity of a complex, several factors have to be taken into account, like the association/dissociation kinetic constants for the prediction of K_D_ as well as the dynamic interactions between the ligands and the proteins. Several studies were performed to predict K_D_ or k_off_ using molecular dynamics simulations ([11,12]). Therefore, the models are only based on partial information; they are single snapshots that, although they capture some experimentally favorable state, may still be incomplete. Since models use only the interactions between the ligands and the proteins, they are generally trained on proteins’ pockets instead of using the whole protein. Pockets have already been calculated for the complexes contained in the PDBbind and are readily available when downloading the database. This removes the need for users to detect new pockets by themselves. Nonetheless, binding affinity will be impacted by conformational information from the ligand and protein local environment [7,13]. Therefore, pockets of different sizes can contain more or less information useful for getting performant models. Here, we investigated the impact of the pocket’s size on the binding affinity prediction.

Other considerations related to the data are also investigated in this study. Notably, the difficulty of predicting the binding affinity of peptides and the impact on the DL model’s performance of using a training dataset including peptides or not. These difficulties stem from the higher degrees of freedom of peptides in comparison to small molecules. This leads to increased complexity of the entropic part when calculating free energies [14]. When training on the PDBbind, it appears that predicting the affinity of peptides becomes a challenging task. Therefore, some published models were developed by training only on nonpeptide ligands [15]. Nonetheless, some nonstructural datasets are specifically designed for antibacterial peptides, and models trained on these datasets have shown good performance [16].

Another aspect pointed out in several recent publications [7,17] is related to DL models memorizing ligand and protein information instead of learning from the interactions. Here, we have deconstructed this by training neural networks only on proteins or ligands and carrying out the prediction to evaluate the bias in their predictions. We compared the performances of three well-known DL models that predict binding affinities—GraphBar, Pafnucy, and OctSurf.

Overall, we find that it is important to train on as much data as possible while even using complexes deemed of lower quality. Moreover, the size of the pocket does matter for the ability of the model to predict the binding affinity. The performance improves upon reaching a certain size (12 Å around the ligand); increasing pocket size further will not improve the performance. On top of this, it is difficult to predict peptides, even by training only on peptides. Finally, we point out that there is a big discrepancy in the ability of neural networks to learn from the interaction. Some models will heavily drop in performance by removing one of the two partners from the complex, while others rely on the memorization of bias in the data to carry out a prediction.

## 2. Results and Discussion

### 2.1. Impact of the Amount of Data on Performance

To reach good performance with DL algorithms, it is expected that more data is beneficial and that a high amount of data is a requirement to begin. In PDBbind (v.2019), the general set contains 17,679 protein–ligand structures. The refined set is a subset of 4852 complexes selected from the general set based on quality criteria. A previously published study suggested that training on the general set of the PDBbind does not improve the performance in comparison to training only on the refined set [7]. However, other studies [18,19,20] pointed out that they achieved better performance by training on the general set rather than only on the refined one.

In order to explore this further, we have trained Pafnucy [10] with the PDBbind general set and with only the refined set. Pafnucy was set up to perform convolutions over voxels of 1 Å^3^ and on a box of 21 Å^3^ centered on the ligand.

**Figure A1:**
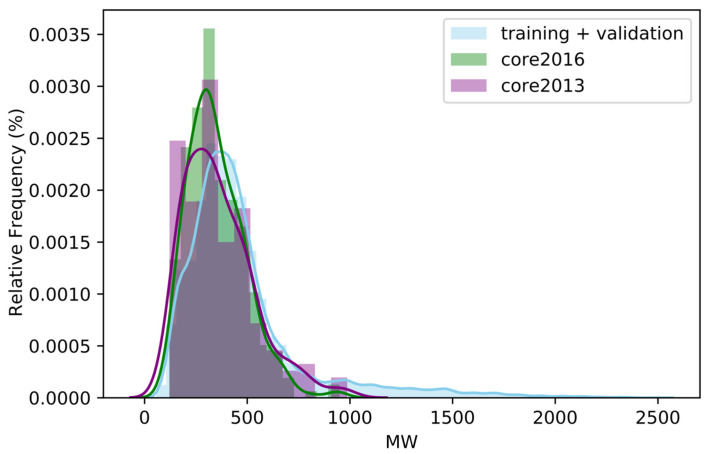
Distribution of PDBbind’s ligands as a function of their molecular weight. The training and validation sets are plotted together in blue, and the test sets are colored in pink and green, corresponding to the core set 2013 and core set 2016, respectively.

**Figure A2:**
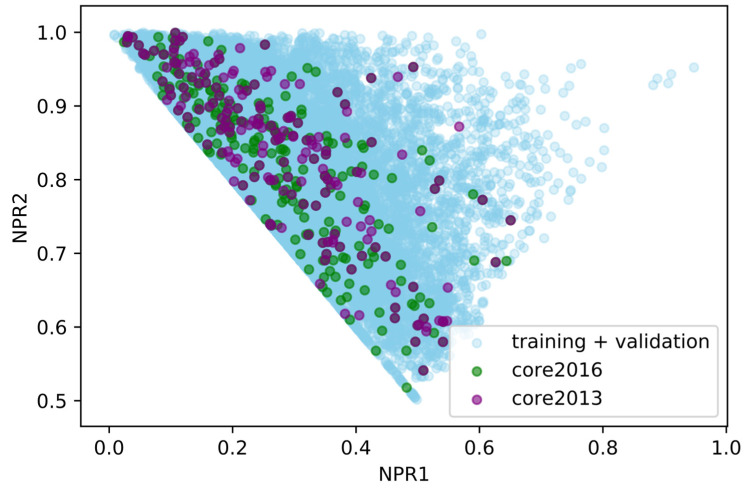
Distribution of PDBbind’s ligands as a function of their shape. The normalized PMI ratio (NPR) is calculated from the principal moment of inertia (PMI) of the ligands. The ligands located at the top right of the plot are spherical, while those at the top left are rod-like. Lastly, the ligands in the bottom of the plot have a disc shape. The training and validation sets are plotted together in blue. The test sets are in pink and green, corresponding to the core set 2013 and core set 2016, respectively.

**Figure A3:**
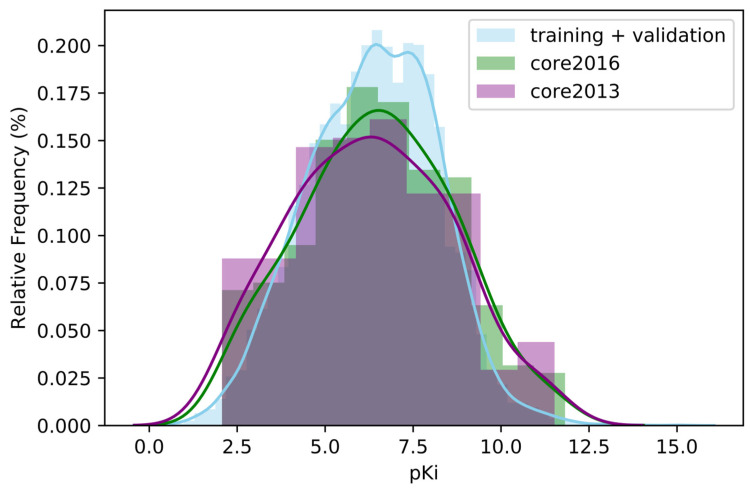
Distribution of the PDBbind’s complexes as a function of their affinity. The training and validation sets are plotted together in blue. The test sets are in pink and green, corresponding to the core set 2013 and core set 2016, respectively.

The models were applied to two test sets comprising 285 and 195 complexes and referred to as core set 2016 and core set 2013. The complexes from the test sets were not used in training. Nonetheless, as reported in GIGN [21], all the proteins and a third of the ligands from the test set are also used in the training set. In other words, none of the test set complexes are present in the training set, but the models have encountered at least one of the binding partners during training. As a result, we can anticipate biased results when making predictions on the test sets. The models might rely on specific data patterns to make predictions. For instance, certain ligands may consistently display either high or low affinity, regardless of the partner protein. This pattern could be exploited by the model, leading to artificially inflated performance. Analyzing these sets further, we found out that the distribution of the molecular weight of ligands is similar for the test set and the training set (Figure A1). The same can be said about the shape of the ligands, although there is a lack of spherical ligands in the test set (Figure A2). In addition, ligands with extreme affinity are over-represented in the test set in comparison to the training set (Figure A3). This can be a possible explanation for why current networks [7] predict over a small affinity range and, therefore, tend to fail at predicting extreme affinity values of the test set.

When assessing performance, we compare the correlation between predicted and experimental activity using the Pearson correlation coefficient (R).

**Figure 1:**
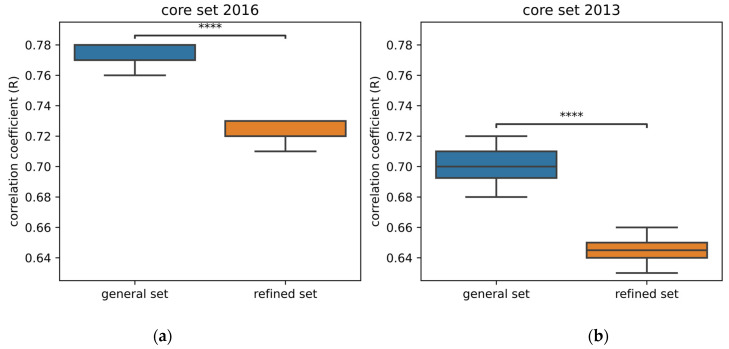
Comparison of the performance of Pafnucy [10] after being trained on the general or the refined set of PDBbind 2019. Ten models were trained on each dataset. (**a**) Performance is evaluated on the core set 2016; (**b**) performance is evaluated on the core set 2013. For both core sets, performance by training on the general set significantly outperforms the performance by training on the refined set. The following *p*-values correspond to the annotations on the plots: ****: *p* ≤ 1.00 × 10^−4^.

Models trained on the general set perform better than those trained on the refined set when applied to the frequently used test sets—core set 2016 and core set 2013 (Figure 1). These results are in accordance with a previously published comparison of the performance of 11 neural networks [18]. For all these neural networks, the RMSE and MAE are lower when trained on the general set instead of the refined set. Likewise, the neural networks PointTransformer [19], DeepAtom [20], and the GNINA CNN v2018 [22] perform better by training on the general set. These results differ a bit with 3D fusion [13], which is a model composed of a 3D-CNN and a spatial graph CNN (SG-CNN). In this case, it seems that 3D-CNN performs better by training on the refined set only, unlike the SG-CNN. Overall, this confirms that having more data, albeit of lower quality, gives better performance. One might question whether the observed performance enhancement obtained by training on the general set can be attributed to proper learning. Did the model develop a more profound comprehension of the interactions or simply enhance its ability to memorize patterns within ligands and proteins?

These results also showcase that there might be a misunderstanding in the field of cheminformatics about the quality of data. Indeed, having data of high quality is very important for carrying out good predictions. Therefore, several teams have decided to train their models only with the refined set, which is considered to be of higher quality. Contrary to that belief, we think that the data that are not in the refined set can still be considered useful data. Indeed, we can compare these data to fuzzy images in image recognition. These images are essential for the robustness of the models in real-life conditions since, in this case, not all images presented to the model would be clear. For image recognition, the saying “garbage in, garbage out” indicates that the images have been badly labeled; therefore, they will impede the training process and result in models with worse performance. In the case of protein–ligand binding affinity predictions, the labeling task of the data has been handled by the team that conceived and updated the PDBbind. They have been manually looking into publications to report the experimentally evaluated binding affinities of complexes [23]. On top of this, the binding affinities obtained were compared to those gathered from MOAD [24], which is another database comprising protein–ligand complexes with binding affinities, in order to reduce the error rate.

### 2.2. Size of Pockets

GNNs are ideally designed to handle the data representing protein–ligand complexes. Indeed, these data are made of nodes (atoms or residues) and bonds (interactions between molecules or intramolecular interactions). Thanks to this design, GNNs focus on the important information, being therefore efficient from a computational point of view. This is not the case for CNNs that are quite computationally intensive, as convolutions are performed on all the voxels of the 3D images. A lot of these voxels do not contain any information about the protein or the ligand, as they are located in the solvent. This increases the calculation time for no performance gain. Although some methodologies have been developed to avoid these hindrances [25], the most common way to reduce the computational requirements while maintaining good performance is to only train models from the pockets instead of using the whole proteins.

The PDBbind provides pockets to the users for convenience. They are constituted of all residues within a distance of 10 Å from the ligand. As the amount of data available for the training increases with the size of the ligand and, therefore, the size of the pockets, we have investigated the influence of the pocket size on the performance of trained models. For this purpose, we have created pockets of different sizes and trained 10 models per size with Pafnucy. We calculated two types of pockets by selecting the residues located within a specific distance measured from all the atoms or from the center of geometry (CoG) of the ligands. The size of pockets was defined by the residue detection distances, ranging from 6 to 14 Å. The size of the box used in Pafnucy is equal to 2 × detection distance + 1 Å^3^.

**Figure 2:**
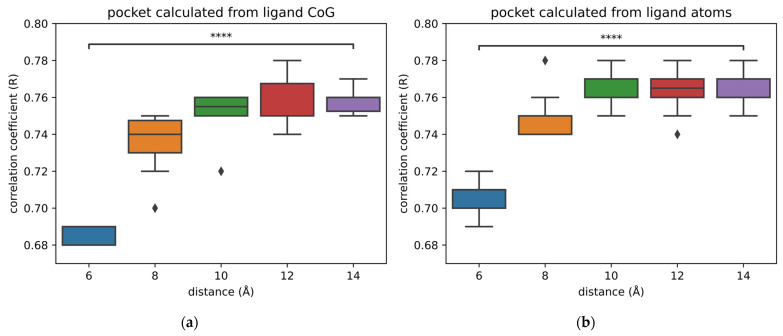
Comparison of the performance of models trained with different sizes and types of pockets. For each size, 10 models were trained and tested on the core set 2016. (**a**) Models trained with pockets made of residues located within a specific distance from the center of geometry of the ligands; (**b**) models trained with pockets created with residues located within a specific distance from all atoms of the ligands. The following *p*-values correspond to the annotations on the plots: ****: *p* ≤ 1.00 × 10^−4^, and ♦ are possible outliers.

For both types of pockets, there is a significant difference in the performance of models trained on pockets of 6 Å and pockets of 14 Å (Figure 2). This is mostly due to the fact that there is more information available in bigger pockets. Therefore, it is advised to use pockets of 10 Å over pockets of 6 Å for training models, regardless of the type of pockets used.

Nonetheless, there is very little improvement in terms of performance between 10 and 14 Å. Thus, there is no interest in using bigger pockets than 10 Å. A compromise is required between using small pockets that do not contain enough information and big pockets that are computationally more expensive while not adding useful information.

Most of the interaction types fall within a range of 6 Å; thus, it is difficult to understand why a pocket size of 6 Å is not sufficient to accurately predict the binding affinity. We think that this can be due to the bias in the data, in which case increasing pocket size and, therefore, adding more amino acids would help the model in memorizing and recognizing patterns in the protein. We could be tempted to think that if we keep increasing the size of pockets, the performance will continuously improve. However, this does not appear to be the case. Hence, there might be a limit to how much the bias in the data can artificially improve the performance.

Apart from the hypothesis of increased bias in the input data, there is an alternative explanation related to the featurization of protein–ligand interactions [26]. Pafnucy describes ligand and protein atoms using 19 atomic features, and the interactions are not explicitly encoded. In this case, the model could detect a hidden influence of amino acids that are not in direct contact with the ligands. Therefore, the model would be able to interpret some long-distance indirect interactions that are not easy for humans to decipher. In this case, the limit in performance reached by using pockets of 10 Å would mean that the amino acids added with bigger pockets are too distant from the ligand to influence it in an indirect fashion. Further investigations are required to confirm or refute these hypotheses. 

Our limitation in interpreting such results is mostly due to the black-box nature of DL algorithms. We do not know the underlying reasons for a given prediction. Using these algorithms on the FEP dataset [27], which contains a chemical series of highly similar molecules targeting the same protein with different affinities, should help in interpreting model performance. Additionally, some methods were developed to alleviate the black-box issue, like layer-wise relevance propagation [28,29], gradient-based methods [30], or masking atoms [31]. Such methods would be useful to better understand the decisions taken by the model that lead to the prediction.

### 2.3. Peptide vs. Nonpeptide

Some neural networks were applied on protein–ligand complexes containing specific types of ligands. PointTransformer [19] was trained on PDBbind 2016, from which 590 complexes labeled as involving peptides were removed.

Ahmed et al. developed a model by training only on proteins in complex with nonpeptides [15]. They created their own dataset by looking into the PDB for protein–ligand complexes with:

- Crystallographic complexes with a resolution lower than 2.5 Å
- Known binding affinity (K_D_/K_i_)
- Ligands that do not have protein chain and are not DNA/RNA

This selection resulted in a dataset of 4041 complexes. By using their neural network called DEELIG, they obtained a model that achieved a correlation coefficient of 0.889 on the PDBbind 2016 core set. These results are encouraging, and it seems worth looking into training models with only peptides and without them.

To evaluate the impact of training only with or without peptides, we flagged the complexes with peptides from the PDBbind. Indeed, among the numerous rules that the PDBbind established in order to select protein–ligand complexes, it has been decided that peptides having 20 residues or fewer would be considered ligands [32]. Therefore, we have detected 2915 complexes interacting with peptides among the 17,679 complexes of the PDBbind (v.2019). 

**Figure 3:**
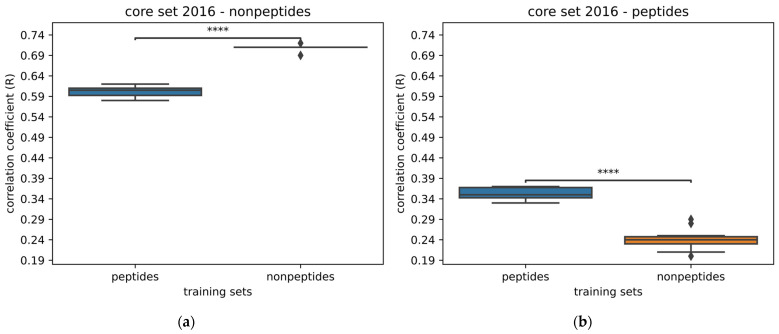
Comparison of the performance of models trained with protein–peptide complexes and with protein–nonpeptide complexes. Models were trained with Pafnucy on 2383 complexes and validated on 492 complexes. (**a**) Performance evaluated on 266 complexes with nonpeptides from the core set 2016; (**b**) performance evaluated on 19 complexes with peptides from the core set 2016. The following *p*-values correspond to the annotations on the plots: ****: *p* ≤ 1.00 × 10^−4^, and ♦ are possible outliers.

**Figure A4:**
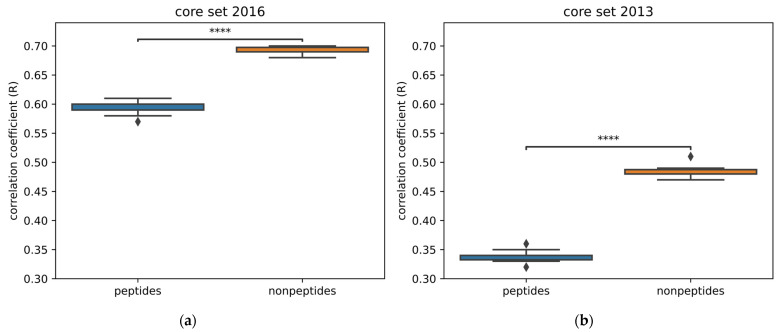
Comparison of the performance of models trained with protein–peptide complexes and with protein–nonpeptide complexes. Models were trained with Pafnucy on 2383 complexes and validated on 492 complexes. (**a**) Performance is evaluated on the core set 2016; (**b**) performance is evaluated on the core set 2013. The following *p*-values correspond to the annotations on the plots: ****: *p* ≤ 1.00 × 10^−4^, and ♦ are possible outliers.

By using Pafnucy, models were trained with complexes interacting with peptides or with complexes interacting with nonpeptides. As the dataset of protein–nonpeptide (PN) complexes is larger than the dataset of protein–peptide (PP) complexes, we randomly subsampled the dataset of PN complexes in order to have datasets of the same size. We trained models by training on each of the even-size datasets. We obtained a model trained on the PN dataset and a model trained on the PP dataset. The performance of models was evaluated on the core set 2013 and 2016 (Figure A4). Performance was significantly better by training on PN complexes. Subsequently, we compared the performance of models by evaluating them on each type of molecule from the core set 2016. Therefore, we tested them only on the PN complexes and only on the PP complexes (Figure 3).

Unsurprisingly, in comparison to the prediction on the whole core set 2016, we see that the prediction gap increases a bit when predicting only on PN complexes. This can also be explained by the fact that all proteins from the PN test set are present in the PN training set, while 40% of them are not in the PP complexes training set. On top of this, 30% of ligands from the PN test set are in the PN training set, and there are none in the PP training set.

As for the prediction carried only on the PP complexes, although the performance of models trained with PP complexes lowers a bit, the drop in performance is more drastic for the model trained on PN complexes. Therefore, it seems that there is information contained in the dataset of PP complexes useful for predicting the PP complexes from the core set 2016, albeit the predictions were carried out on only 19 complexes. We can point out that 50% of the ligands are in the PP training set, while none are in the PN training set.

**Figure 4:**
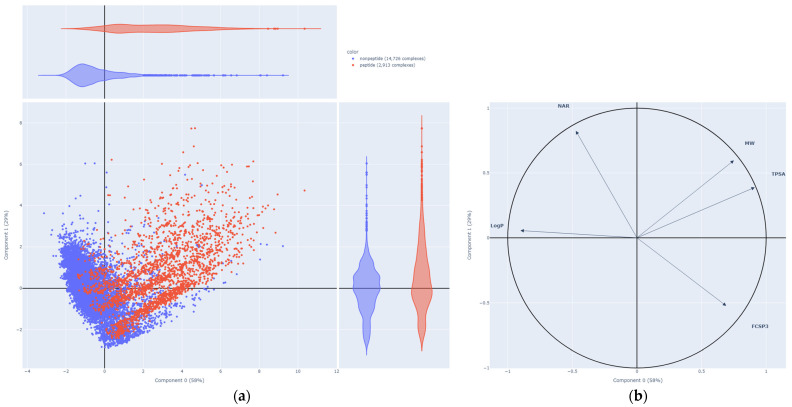
Principal component analysis applied on the ligands of the PDBbind dataset. Peptides are colored in red, while the rest of the ligands are displayed in blue. (**a**) Plot of the individuals; (**b**) correlation circle.

We explored the chemical space of the PDBbind to better understand the difference in performance between models trained on PN and PP complexes by performing a principal component analysis (PCA) on the ligand of the complexes from the PDBbind dataset. This allows us to compare the distribution of peptide and nonpeptide ligands (Figure 4). The descriptors used to characterize the ligands were selected based on the literature [33], then the correlated descriptors were removed. The following five descriptors were used to carry out the PCA: hydrophobicity (LogP), Topological Polar Surface Area (TPSA), Fraction of SP3 hybridized Carbon (FCSP3), Number of Aromatic Rings (NAR), and Molecular Weight (MW).

The PCA displays 87% of the variance of the data. It appears that the two populations of ligands are well separated. These results showcase the difference between peptides and small molecules, which helps explain the lower performance from training with complexes involving only one type of ligand and predicting on the other type. Furthermore, the peptides are known to have high degrees of freedom, especially due to the peptide bonds [14]. This increased flexibility results in a high level of entropic energy, which needs to be taken into account when carrying out free energy prediction. Consequently, the evaluation of such values is very challenging. This can be an explanation for the poor performance of models in predicting the binding affinity for PP complexes.

**Figure A5:**
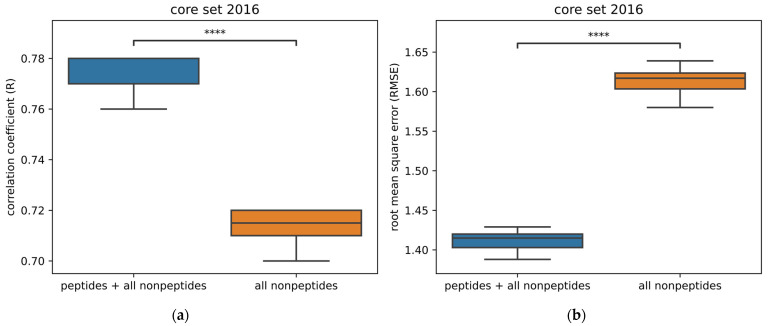
Comparison of the performance of models trained on the whole PDBbind and with only protein–nonpeptide complexes (trained on 13,403 complexes and validated on 1000 complexes). Models were trained with Pafnucy. Performance is evaluated on the core set 2016 (285 complexes). (**a**) Performance is evaluated with the correlation coefficient; (**b**) performance is evaluated with the root mean square error (RMSE). The following *p*-values correspond to the annotations on the plots: ****: *p* ≤ 1.00 × 10^−4^.

We also evaluated the performance of models trained only on PN complexes in comparison to training with both ligands mixed. Contrary to what we expected, it seems that training only on PN complexes does not improve the performance of the models (Figure A5). This comes as a surprise, as we anticipated obtaining better performance in a similar fashion to DEELIG [15]. An explanation for the very high performance (R = 0.889) obtained by DEELIG is that 68% of the test set complexes were used for the training, therefore skewing the evaluation of performance.

Nonetheless, even if it is better to train on the maximum amount of data possible, there are promises to develop some local models focused on specific types of ligands. This is a practice less common than creating local models based on the type of proteins involved, but it can lead to interesting results. Moreover, it would be worth investigating transfer learning in such cases. For example, general models would be developed by learning general rules on the maximum amount of data and then be specialized in predicting the binding affinities of peptides, for example.

Once again, these results should be interpreted with caution, as there are strong indications of bias in the test set. For example, as we pointed out previously, all the protein families from the test are also present in the training set. The same issue applies to the ligands from the test set, with at least 30% of them also being in the training sets but bound to different proteins.

### 2.4. Replication of Results

Most neural networks are nondeterministic. This behavior leads to variation in the performance of models trained with the same neural network and the same data. Indeed, several factors influence the variability, one of them being that initial weights are assigned randomly across the neural network at the beginning of the training. Due to the randomized assignment of weights, the model is more likely to fall into certain local minima, creating uncertainty for the estimation. One way to overcome this issue is to modify the learning rate during the training by using a learning rate scheduler and, therefore, getting out of local minima. The other solution is to train several model replicates to increase the chances of having a model that does not fall into a local minimum. In any case, it is still necessary to carry out ensemble approaches [34] in order to accurately evaluate model performance and replicability. This implies training several models, averaging their performance, and evaluating the standard deviation. This was performed in the publication of OctSurf, where each value was averaged from five models. For this study, we replicated the results of three neural networks (Pafnucy [10], GraphBAR [35], and OctSurf [25]) and evaluated their averaged performance by training 10 models each time (Table 1).

We were able to reproduce the performance displayed in the publication of each neural network.

All the standard deviations (SDs) have low values like 0.01 or 0.02. Nonetheless, an SD of 0.02 means that, with GraphBAR, it is as likely to get models with a correlation coefficient of 0.74 as 0.78 on a similar test set. As this is a relatively big difference in terms of performance, we think that deep ensemble averaging [36] should always be applied when publishing the results of training models with a neural network. Although this is computationally intensive, it gives more reliable expectations for people reusing the same neural network, as well as preventing bias like selecting the best model and publishing its results as representative of the neural network performance.

Another use of model replicates is to build ensemble models. Instead of measuring the correlation coefficient for each model and calculating the mean and the standard deviation, it is possible to calculate the mean prediction for each sample and then calculate the correlation coefficient. This methodology has already been applied for several deep learning models like PIGNet [37] and in Francoeur et al. [22]. It leads to a small gain in performance; for example, by using this methodology, Pafnucy and GraphBAR obtain an R = 0.79. As for their RMSE, Pafnucy improves from 1.41 to 1.38 and GraphBAR from 1.43 to 1.37. Such consensus methods are, therefore, a good way of improving performance while being less subject to variations.

### 2.5. Learning from Ligand Only, Protein Only, or Interactions

Achieving good performance on a test set is the primary goal in model development, but it is also necessary to verify if such high performance is not due to learnt biases from the data. As mentioned previously, the PDBbind core set is heavily biased, with both proteins (all) and ligands (~30%) represented in the training set. Therefore, models will tend to shortcut learning by using easily learnable biases that might not be present in other datasets. This is what is called a noncausal bias, where there is correlation but no causation. As mentioned by Sieg et al. [38], models can artificially achieve good predictions by learning patterns that are not related to meaningful physicochemical mechanisms for binding. For example, it appears that most of the reported binding affinity prediction models only memorize ligand and protein information instead of learning from their interactions [7]. This appears to be a major issue in the field, as it leads to poor generalization power.

A number of strategies have been suggested to compel neural networks to learn from interactions for virtual screening purposes [39,40]. For instance, decoy poses have been generated by modifying the position of ligands. These decoy poses were obtained by redocking active compounds and selecting a low-energy pose with a high RMSD from the initial position. Even simpler methods, like rotating and translating the ligands, have been applied. In a similar way, we propose that this could be applied on the PDBbind dataset by either redocking, rotating, or translating high-affinity ligands. The resulting decoy poses would be labeled with low affinity. Consequently, when trained on such datasets, models will encounter several occurrences of the same complexes with different ligand positions and different binding affinities. Therefore, we anticipate that these models could adapt from primarily performing QSAR to potentially gaining a deeper comprehension of protein–ligand interactions. Previous works were published on the topic of data augmentation with docking for scoring functions [22,35,41,42]. To the best of our knowledge, all of them focused on selecting poses similar to the crystallographic one and assigning similar binding affinities. Another idea would be to dock ligands with low affinity from the CHEMBL, especially the ones that are structurally similar to high-affinity ligands from the PDBbind. In the case that these ligands interact with the same proteins, we would add the notion of activity cliff to the models. These data augmentation methods would help the models generalize by making them focus on the interactions rather than memorizing the bias inside the dataset. However, it is essential to exercise caution when combining experimental and synthetic data. We have not used the aforementioned methods in this study, and we will discuss this in more detail in future work.

**Figure 5:**
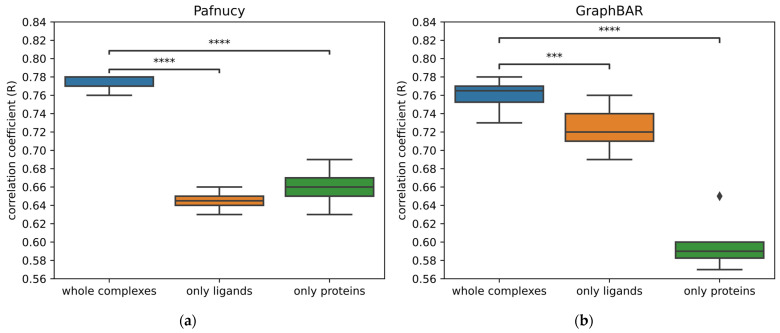
Comparison of the performance of Pafnucy and GraphBAR without either the protein or the ligand. The performance of models was evaluated on the core set 2016. Learning on the whole complexes leads to significantly better performance. (**a**) The mean prediction gap between training on whole complexes or training on ligands alone is at 0.12 of coefficient correlation for Pafnucy, while (**b**) it is only at 0.03 for GraphBAR by training only on the ligands. The following *p*-values correspond to the annotations on the plots: ***: 1.00 × 10^−4^ < *p* ≤ 1.00 × 10^−3^, ****: *p* ≤ 1.00 × 10^−4^, and ♦ are possible outliers.

As mentioned previously, there are several visualization tools that reveal which parts of a structure are important when carrying out a prediction. In Hochuli et al. [31], those methods were applied on GNINA CNN v2017 [40] in order to understand its underlying reasoning for the classification of active and inactive molecules. Another way to uncover if a model truly learnt from the protein–ligand interactions is to train other models by removing either the protein or the ligand. Subsequently, the models trained on partial data are evaluated on the test set with the same partners removed. This evaluation helps us understand the performance difference between learning and predicting with the entire complex compared to learning and predicting with only the ligand or protein. To facilitate this comparison, we calculate the prediction gap between learning on the full complexes and learning on one of the two partners. The bigger the gap in prediction is, the better the model’s understanding of the interactions. However, these considerations are relatively recent. Only a few neural networks have been evaluated for their ability to learn from interactions and not only memorize structural patterns in proteins or ligands. For this purpose, in Figure 5, we have evaluated the ability of learning on interactions for two already published neural networks: a convolutional neural network (Pafnucy) and a graph convolutional neural network (GraphBAR).

With both neural networks, training on the whole complexes gives significantly better performance than training on the ligand or protein structures alone. Nonetheless, we can see disparities between the two neural networks, as the difference in the correlation coefficient by training only on the ligands compared to the whole complexes is 0.12 for Pafnucy, while it is only at 0.03 for GraphBAR. This means that Pafnucy does a better job at analyzing the interactions made between the proteins and the ligands, while GraphBAR seems to rely more heavily on learning patterns from ligands and then correlating them to binding affinities.

In the publication of OctSurf, the performance was also evaluated by training only on ligands and only on proteins. A correlation coefficient of 0.79 was reported for the full complex, while reaching 0.73 with ligands and 0.65 with proteins. Thus, the prediction gap is at 0.06, which is between Pafnucy and GraphBAR.

Other binding affinity models have been tested for their ability to learn from the interactions by training only on proteins or only on ligands. All these results have been summarized in Table 2. The results of the Modular MPNN [7] are in accordance with previously evaluated neural networks. Nonetheless, Deep Fusion [13] and PointTransformer [19] achieve a bigger prediction gap by removing either the ligand or the protein. This goes up to 0.41 for PointTransformer when learning only on ligands.

From these results, it seems that the ability of neural networks to learn from the interactions can vary importantly. The PDBbind 2019 was used as training data for both Pafnucy and GraphBAR, and both used similar descriptions of atoms. Therefore, the main factors differentiating the two are the underlying structure of the networks and the ensuing way of handling the data and carrying out prediction.

Accordingly, Deep Fusion reuses the same preparation protocol as Pafnucy in terms of atomic description, for example. Furthermore, it combines a 3D-CNN and a spatial graph CNN; this unique approach might be the reason for the model’s ability to better understand the protein–ligand interactions.

PointTransformer is a point cloud-based neural network, like OctSurf. Therefore, we expected this tool to have a similar prediction gap to OctSurf. On the contrary, the prediction gap was much more important with PointTransformer.

### 2.6. Other Test Sets

As shown throughout this paper, there are numerous biases contained in the core sets from the PDBbind. Due to this, we think it is important to use other types of benchmark datasets to accurately validate the new models developed. Indeed, the evaluation of models across several test sets grants a higher confidence when comparing performance. Across time, several other test sets have been developed to evaluate the scoring and ranking power of models. The scoring and ranking power are, respectively, the model’s ability to accurately predict the binding affinity and its ability to correctly rank ligands by using the predicted binding affinity.

There are test datasets that have already been used in numerous publications [2]. For example, the Astex diverse set [43] was used to validate Pafnucy [10], DeepAtom [20], and RosENet [44]. It includes 85 protein–ligand complexes, 74 of which have known binding affinity. There are, as well, other test sets called the CSAR-NCS HiQ set 1 and set 2 [45], which are composed of 176 and 167 complexes from the Binding MOAD [24] and the PDBbind. After removing the complexes overlapping with the usual training set, around 50 and 40 complexes remain for both test sets (Table A1). They have been used to evaluate K_DEEP_ [46], RosENet [44], OnionNet-2 [47], graphDelta [48], GraphBAR [35], PIGNet [37], BAPA [49], CAPLA [50], and GIGN [21].

The FEP dataset [27] originally used in free-energy perturbation studies has also been applied to evaluate the binding affinity predictions of several models [44,46,48]. It is used to test the ability of a model to discriminate between several similar ligands with different binding affinities for the same protein. It is composed of eight proteins: BACE, CDK2, JNK1, MCL1, p38, PTP1B, Thrombin, and Tyk2. Each protein family is represented by one structure. There are 200 ligands obtained from a small number of scaffolds. Their 3D positions in the binding site are provided, and their affinities have been obtained experimentally. This information is summarized in Table A2.

Holdout test sets have also been developed to evaluate the performance of models on recent data. These test sets are obtained by performing a temporal split over a dataset, i.e., training models on complexes released before a specific date and testing them on complexes released afterward. The holdout test sets are generally large, with complexes that were not cherry-picked and thus are less likely to be biased.

- An example of such a dataset can be found in Volkov et al. [7], where a modular MPNN and Pafnucy were trained on the PDBbind 2016 and were evaluated by predicting on a 2019 holdout set. To create this test set, they selected 3,386 complexes from the PDBbind 2019 that are not in the PDBbind 2016. Instead of using the files provided by the PDBbind, they downloaded the structures from the Protein Data Bank [51]. The complexes were curated and processed with Protoss v.4.0 [52] and IChem [53], e.g., protonation was optimized. Subsequently, Isert et al. [54] reused these data to train models with electron density-based geometric neural networks, and they validated their binding affinity predictions on the same 2019 holdout set.
- Another 2019 holdout set of 4,366 complexes was used to evaluate GIGN [21]. They compared their results against a dozen neural networks, including OnionNet [55], Pafnucy, and GNN-DTI [56]. It is worth mentioning that the protein overlap rate between test and training sets is 69% instead of 100% for the core set 2016. As for the ligand overlap rate, it goes down to 25%, while it was at 38% for the core set 2016.
- Due to similar considerations, Deep Fusion [13] was evaluated on a test set of 222 complexes that was developed from the 2019 holdout set by removing complexes with ligands or proteins already present in the PDBbind 2016. Deep Fusion, K_DEEP_, and Pafnucy were trained on the PDBbind 2016 and evaluated on this test set.
- AK-score [57] was trained on the refined set of the PDBbind 2016, and it was evaluated by predicting the binding affinity of 534 complexes newly released in the refined set of the PDBbind 2018. For comparison purposes, they also evaluated the performance of other scoring functions, namely X-score [58] and ChemPLP [59].
- The atomic convolutional neural network (ACNN) [60] was trained and tested on several different splits of the PDBbind dataset. On top of a temporal split, they used a stratified split based on the pK_i_ value of complexes and a ligand scaffold split. The stratified split allowed the selection of complexes covering all binding affinities in the training and test sets. In the case of the scaffold split, ligands with unusual scaffold were placed in the test set, therefore preventing the effect of QSAR in the prediction.
- In a similar way, MoleculeNet [61] has been trained and tested on the PDBbind dataset with a temporal split. As for PotentialNet [62], they performed cross-validation by performing a pairwise structural homology split and a sequence similarity split. Both splits are explained in detail in Li and Yang [63]. They were carried out via an agglomerative hierarchical clustering on the PDBbind 2007 refined set, resulting in a test set of 118 and 101 samples, respectively.

The PDE10A dataset [64] has been recently released, with 1,162 docked or co-crystalized PDE10A inhibitors. These data are sourced from a former project of Roche; thus, the binding affinities (IC50) were obtained in a consistent way. There are 77 PDE10A complex structures obtained by crystallography, and the rest of the complexes were generated through multi-template docking. The test sets were obtained by using temporal and binding mode splits. There are three temporal split test sets: the 2011, 2012, and 2013 test sets, with 250, 141, and 73 complexes, respectively. Similarly, there are three binding mode split test sets: the aminohetaryl_c1_amide, c1_hetaryl_alkyl_c2_hetaryl, and the aryl_c1_amide_c2_hetaryl test sets, composed of 452, 291, and 419 complexes, respectively. They compared their 2D3D ML methods against PotentialNet [62] and ACNN [60]. Isert et al. [54] also benchmarked their neural networks on these test sets.

Apart from the scoring and the ranking power, there are other criteria that can be used to evaluate drug–target interaction models, like the virtual screening (VS) power. This criterion defines the ability of a model to discriminate between decoys and active molecules. As brought up in PIGNet [37], in order to accurately assess the performance of a model, it is advised to evaluate not only its scoring power but also its virtual screening power. For evaluating such ability, datasets incorporating decoys have also been used as test sets. Nonetheless, warnings must be raised about using these datasets. Indeed, most of them are also biased [38], especially when splitting one of them into training and test sets, which usually leads the underlying models to achieve artificially high performance. On the contrary, when training a scoring function on the PDBbind and predicting on vs. datasets, the results are usually lower. The performance of models evaluated on vs. datasets is measured by calculating the area under the ROC curve (AUC), which increases when active molecules are predicted with higher binding affinities than decoys. Furthermore, it is possible to evaluate scoring functions by calculating the enrichment factor (EF) from the ROC curve. The EF is obtained by measuring the true-positive rate (TPR) for a given false-positive rate (FPR). Therefore, it is possible to evaluate the model’s ability to find active molecules over decoys for its best-scored docking poses. Hence, the EF is more representative of the use of vs. tools in real conditions, as users are mostly interested in the ligands with the highest score.

Examples of such datasets are the DUD [65] (directory of useful decoys) and DUD-E (enhanced DUD) [66]. They are used for benchmarking molecular docking by providing active molecules and decoys (assumed inactive) for given targets. They have been developed to deal with the usual dataset problems, like “artificial enrichment”, which corresponds to having decoys that are very different from active molecules, and “false negative bias”, referring to decoys turning out to be active after being tested experimentally. The DUD-E is an enhanced version of the DUD with an increased amount of data. It is designed to address the “analogue bias” of having highly similar active molecules. The DUD and DUD-E are composed of 2950 and 22,886 active molecules, respectively, as well as 95,326 and 1,411,214 decoys (up to 50 decoys per active molecule charge states) for 40 and 102 targets. Unfortunately, there are still biases present in the DUD-E [67]. Especially, an analogue bias intra- and intertarget was detected. These biases add up with the decoy bias, which is the similarity of a decoy from the same target. When trained on a part of the DUD-E and evaluated on the other part, models obtain the same high performance (AUC > 0.9) if we keep the whole complexes or only use the structure of the ligand. Therefore, this leads to similar issues as the ones related to the PDBbind core set.

- The DUD-E was used to train AtomNet [68] and to evaluate its virtual screening power. AtomNet is the first CNN applied on 3D grids to predict protein–ligand binding affinities. Thirty targets from DUD-E were used as the test set, while the remaining seventy-two targets were used as the training set. On top of using the DUD-E dataset, a derived dataset called “ChEMBL-20 PMD” has been compiled to further benchmark AtomNet. It was created based on several quality criteria and is composed of 78,904 actives, 2,367,120 property-matched decoys (PMD), and 290 targets. This dataset is composed of decoys structurally different from the active molecules to prevent the false-negative bias issue, which, on the other hand, results in an artificial enrichment issue. Therefore, another dataset called “ChEMBL-20 inactives” was developed in order to evaluate AtomNet’s ability to classify experimentally verified active and inactive molecules. ChEMBL-20 inactives were obtained by replacing the PMD with 363,187 molecules known to be inactive.
- Lim et al. [56] used the DUD-E and the PDBbind in order to constitute a training set and a test set. Molecules were docked with Smina [69], resulting in a dataset of docked poses for DUD-E’s 21,705 active molecules and 1,337,409 decoys. As for PDBbind, the molecules were redocked with Smina. If the pose had an RMSD < 2 Å from the crystallographic pose, it was classified as a positive sample, and if the pose was at >4 Å from the crystallographic pose, it was classified as a negative sample. Therefore, 2094 positive and 12,246 negative samples were obtained. The training set was subsequently created with the docked poses of 72 proteins from the DUDE and 70% of the PDBbind redocked dataset. The test set consisted of the docked poses from the remaining 25 proteins from the DUDE and 30% of the PDBbind redocked dataset. The PDBbind split of data was based on a split of the targets, so no proteins would be in the training and test sets. Thereafter, another test set was developed by selecting from the CHEMBL molecules with known binding affinity for the 25 proteins from the DUDE test set. The affinity threshold was put to an IC50 of 1.0 μM, splitting the test set into 27,389 active and 26,939 inactive molecules.

Similar to the DUD/DUD-E, the DEKOIS 2.0 [70] dataset was developed to evaluate scoring functions for their virtual screening power. It is composed of 81 benchmark sets for 80 protein targets (one target having two different binding sites and benchmark sets). There are 40 active molecules per benchmark set. For each active molecule, 30 structurally diverse decoys were selected, resulting in 1200 decoys per benchmark set. The DEKOIS dataset is constituted of decoys that have not been tested experimentally; therefore, decoys were selected by matching the properties of the active molecules in order to avoid artificial enrichment. Furthermore, the selection of the decoy has been tailored to prevent the occurrence of latent actives in the decoy set (LADS). LADS are molecules supposed to be decoys, which actually have an activity for the target. This issue was previously referred to in the study as false-negative bias. Only 4 targets out of the 81 of the DEKOIS dataset are in common with the DUD-E [71], but 26 targets have at least 95% sequence identity with DUD-E targets [72]. As pointed out in the Ballester paper [73], several machine-learning scoring functions [71,72,74] were trained on DUD-E and evaluated on DEKOIS.

The Maximum unbiased validation (MUV) is another dataset developed to benchmark virtual screening tools. It is composed of active and inactive molecules experimentally tested for 17 target proteins. For each target protein, there are 30 actives and 15,000 decoys with known binding affinities. In a similar fashion, Riniker and Landrum [75] created a dataset from CHEMBL comprising 50 targets, with 100 diverse active molecules per target and two decoys per active molecule, leading to 10,000 decoys. The GNN-DTI from Lim et al. [56] was evaluated on the MUV dataset. GNINA CNN v2017 [40] and the DenseNet CNN from Imrie et al. [76] were evaluated on a part of both the MUV dataset and the ChEMBL dataset from Riniker and Landrum. The active molecules and decoys were docked with Smina [69] or AutoDock [77]. For the MUV dataset, out of the 17 target proteins, 9 were used in the test set. Therefore, this led to 1913 poses associated with the 270 active molecules and 1,177,989 poses associated with the 135,000 decoys. As for the CHEMBL dataset, 13 targets among the 50 targets were used, leading to 11,406 poses associated with 1300 active compounds and 663,671 poses associated with 10,000 decoys.

In the CASF update [78], the scoring power, the ranking power, the docking power, and the screening power of several scoring functions were evaluated on the core set 2016. The docking power corresponds to the ability of a scoring function to identify the native ligand binding pose among several decoy poses of the same ligand. More than 30 scoring functions were evaluated for these criteria.

- To assess the docking power, decoy poses were generated by redocking PDBbind’s ligands in their binding site. For each complex, up to 100 decoy poses were selected by setting up 10 bins of 1 Å based on their RMSD values (0–10 Å) to the initial pose. For each bin, ligand poses were clustered based on their conformation, and up to 10 poses were selected. This led to a dataset composed of 22,492 decoy poses.
- In order to evaluate virtual screening power, the ligands were crossdocked. The dataset is composed of 16,245 protein–ligand interaction pairs by docking 285 ligands into 57 proteins. The docking was performed on the protein structure with the highest affinity for each cluster. One hundred poses were selected for each protein–ligand interaction pair. Overall, 1,624,500 decoy poses make up this dataset.

In Francoeur et al. [22], several docking datasets have been compiled in order to train and test their neural networks. They obtained a test set of 4618 poses by redocking 280 complexes from the PDBbind core set 2016 and selecting up to 20 poses per complex. In a similar fashion, they redocked 3805 complexes from the refined set and 11,324 from the general set, leading to 66,953 and 201,839 poses, respectively. Thereafter, they created the CrossDocked2020 dataset by crossdocking complexes from the Protein Data Bank [51] that were selected based on the similarity of the binding pockets. They trained their neural networks on a first version of this dataset, then selected wrongly predicted poses as data augmentation for retraining the model. This iterative reinforcement learning method led to a dataset of 22,584,102 poses (786,960 redocked poses and 21,797,142 crossdocked poses) from 18,450 complexes. Forty-two percent of these complexes have known binding affinities from the PDBbind. From there, the BigBind dataset [79] was created by mapping CHEMBL activities to the 3D structures of protein pockets in CrossDocked. By doing so, the number of pockets was reduced from 2922 (in CrossDocked2020) to 1067. The resulting dataset contains 11,430 3D structures, with 851,359 activities spanning 531,560 unique compounds.

In the GNINA CNN v2017 publication [40], the docking power was evaluated by redocking the 2013 PDBbind core (195 complexes). They obtained 98 low RMSD poses (<2 Å from the crystallographic pose) among a total of 897 poses. The training was carried out on redocked complexes from the CSAR-NRC HiQ data set [45] and the CSAR HiQ Update. From the initial 466 complexes, they redocked 377 complexes having a binding affinity > 5 pK. Poses at less than 2 Å from crystallographic poses were labeled as positive, while those at more than 4 Å were labelled as negative. Those between 2 and 4 Å were discarded. This led to a dataset composed of 745 positive poses (from 327 complexes) and 3251 negative poses (from 300 complexes).

Famous datasets like the PDBbind/CASF, the DUD-E, or the MUV have been applied to train and evaluate many models. Unfortunately, it appears that most of the famous datasets are biased. Although they may still be relevant to some extent for comparison purposes, we have seen the development of a myriad of new benchmark datasets. Many papers presenting new neural networks demonstrated their performance on custom test sets. For example, six papers developed their own training and test sets by performing a temporal split. For a better comparison of models, it would be preferable to evaluate their performance on a common benchmark dataset obtained through temporal split.

Overall, we think that it is important to evaluate the scoring power of models on several benchmark datasets to get an accurate evaluation of their performance. On top of that, we advise the evaluation of their ranking, docking, and screening powers. By doing so, we can get a better idea of their usefulness in real-case scenarios.

## 3. Materials and Methods

### 3.1. Datasets

The PDBbind dataset (http://www.pdbbind.org.cn (accessed on 8 November 2023)) [9] was used to train the different models. It contains protein–ligand complexes with known binding activity. In its current version (v.2020), 19,443 complexes are available. In this publication, three versions of the PDBbind were used:

- The version 2016 that contains 13,308 protein–ligand complexes
- The version 2018 that contains 16,151 protein–ligand complexes
- The version 2019 that contains 17,679 protein–ligand complexes

The complexes present in the PDBbind are selected from the Protein Data Bank (https://www.rcsb.org/ (accessed on 8 November 2023)) [51]. Several modifications are added to these complexes, e.g., the biological assembly of complexes is recreated, and ligands’ atoms and bonds are corrected; for details of all modifications, please refer to the “readme” provided with the PDBbind.

The PDBbind encompasses three sets of data: the general set, the refined set, and the core set. The general set contains the totality of the dataset. The refined set is a subset made of 4852 complexes (for the version 2019) selected on the basis of the following quality criteria [80]:

- Crystallographic structures, with a resolution of 2.5 Å maximum
- Complete ligands/pockets (without missing atoms) and without steric clash with the protein
- Noncovalently bound complexes, no nonstandard residues at a distance <5 Å from the ligand
- No other ligands are present in the binding site, e.g., cofactors or substrates
- Binding affinity evaluated in K_i_ or K_D_ and with a pK_i_ between 2 and 12
- Ligands with a molecular weight of less than 1000 and less than 10 residues for peptides
- With ligands made only of the following atoms: C, N, O, P, S, F, Cl, Br, I, and H
- The buried surface area of the ligand is higher than 15% of the total surface area of the complex

The core set is broadly used as a test set to compare models’ performance. Only two versions are available: version 2013, which is composed of 195 complexes [81,82], and version 2016, comprising 285 complexes [78]. Both core sets have 107 complexes in common. The core set 2016 is made of 57 clusters of 5 complexes belonging to the same protein family. These groups are obtained by clustering complexes based on sequence similarity of 90% minimum.

In this study, peptides were flagged among the ligands coming from PDBbind’s complexes. We detected the peptides by looking for ligands having in their mol2 files at least one atom named “CA”, “CB”, “CD”, “CE”, “CG”, “CZ”, “CA1”, “CA2”, “CB1”, “CB2”, “CD1”, “CD2”, “CE1”, “CE2”, “CG1”, “CG2”, “CZ1”, or “CZ2”. On top of this, we analyzed the PDBbind list of ligand names and flagged as peptides all the ligands containing “mer” in their name. Finally, ligands wrongly labeled as peptides were removed by keeping only ligands matching with the following smart, which represents a peptide bond: [$([NX3H2,NX4H3+]),$([NX3H](C)(C))][CX4H]([*])[CX3](=[OX1])[OX2H,OX1-,N]. By doing so, we were able to detect 2915 peptides in the PDBbind (v.2019). The list of peptides curated from the PDBbind v.2019 was made available as supplementary material (Table S1).

We used the pockets provided by the PDBbind to evaluate the impact on the performance of:

- The dataset sizes (general set or refined set)
- The types of ligands (peptide or nonpeptide)
- Using only ligands or only proteins

**Figure 6:**
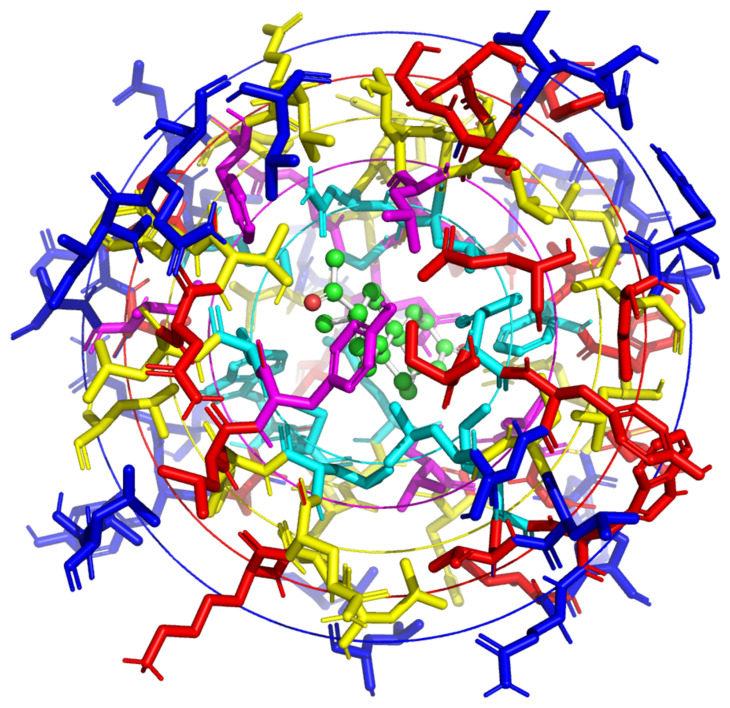
Pockets created and visualized with Pymol. The ligand is displayed in green. Residues are colored in cyan, purple, yellow, red, and blue according to their distance from the CoG of the ligand, at 6 Å, 8 Å, 10 Å, 12 Å, and 14 Å, respectively.

We also created our own pockets using Pymol. Residues around the ligands were selected to create pockets. The pockets were constructed with different sizes: 6 Å, 8 Å, 10 Å, 12 Å, and 14 Å. Two types of pockets were created by selecting residues at a specific distance from:

- All the atoms of the ligands
- The center of geometry (CoG) of the ligands (Figure 6)

### 3.2. Neural Networks

Protein–ligand complexes can be used to train statistical models in many ways. The 3D representations of these complexes can be either 3D structures or 3D surfaces [83], which can be implemented in various ways, including 3D grids, point clouds, 3D graphs, or mesh [83,84]. Several types of neural networks were developed to handle these representations of the data, such as convolutional neural networks (CNNs) and graph neural networks (GNNs). CNNs are used on 3D grids that discretize the space in voxels of around 1 Å^3^. Then, CNNs perform convolutions over these voxels to extract meaningful information for the prediction of binding affinities. GNNs are applied on graphs, where atoms serve as nodes and bonds as edges. In the case of graph convolutional networks, the useful information stored in nodes and edges is extracted by performing graph convolutions.

Only previously published binding affinity neural network approaches were used in this work. For the purpose of this study, we selected two CNNs: Pafnucy [10] and OctSurf [25], both employing grids to discretize 3D structures and 3D surfaces, respectively. Additionally, we evaluated GraphBAR [35], which is a graph convolutional neural network. Here, we briefly describe each of them. The full description of the neural networks can be found in the original publications.

Pafnucy is a 3D convolutional neural network published in 2018. It uses the 3D coordinates of atoms and performs convolutions on voxels of 1 Å^3^. In this paper, we generally used boxes of 21 Å and modified the size of the box when different sizes of pockets were used. Nineteen features were used to describe an atom:

- 9 bits (one-hot or all null) encoding atom types: B, C, N, O, P, S, Se, halogen, and metal
- 1 integer (1, 2, or 3) with atom hybridization: hyb
- 1 integer counting the numbers of bonds with other heavyatoms: heavy_valence
- 1 integer counting the numbers of bonds with other heteroatoms: hetero_valence
- 5 bits (1 if present) encoding properties defined with SMARTS patterns: hydrophobic, aromatic, acceptor, donor, and ring
- 1 float with partial charge: partial charge
- 1 integer (1 for ligand, −1 for protein) to distinguish between the two molecules: moltype

This neural network uses data augmentation by learning from systematic rotations of complexes. The systematic rotations are obtained by performing the 24 rotations of the cube on each structure. The data augmentation with systematic rotations allows the models to be more robust since the models are independent of the orientations of the ligands and the proteins.

Here is the reported performance of Pafnucy trained on the pockets provided by the PDBbind 2016:

- Core set 2013: correlation coefficient of 0.70 taken from Stepniewska-Dziubinska et al. [10].
- Core set 2016: correlation coefficient of 0.78 taken from Stepniewska-Dziubinska et al. [10].

We replicated the results of Pafnucy by using the code available here: https://gitlab.com/cheminfIBB/pafnucy (accessed on 8 November 2023).

OctSurf is a 3D convolutional neural network published in 2021. It requires elaborate data preparation before it can be used as input for the neural network. First, the 3D coordinates of atoms are turned into point clouds [85] representing their van der Waals surfaces. Then, the point clouds are rasterized into an octsurf, which is a volumetric representation based on an octree data structure [86]. An octsurf is composed of octants on which the convolutions are performed. The octants can have variable sizes. This allows for having octants of different sizes in the same octsurf, describing more or less precisely different parts of the octsurf. Therefore, it is possible to have big octants in the solvent and smaller ones (of 1 Å, for example) in contact with the proteins and ligands. This way, we can accelerate the convolution process while keeping good performance.

The description of octants uses the 19 features described in Pafnucy. On top of that, 5 more features were added to reach a total of 24 features:

- The hydrogen atom type
- van der Waals atomic radius
- A normal vector with three coordinate directions describing surface curvature and shape complementarity

Data augmentation was performed by randomly rotating and translating the surface points, reaching 40 octsurfs for each complex.

In the publication, OctSurf reached a correlation coefficient of 0.79 [25] on the core set 2016 by training on the pockets provided by the PDBbind 2018.

The code of OctSurf is available here: https://github.uconn.edu/mldrugdiscovery/OctSurf (accessed on 8 November 2023).

GraphBAR is a graph convolutional neural network published in 2021. Graphs were created with atoms as nodes and bonds as edges. Node characterization reuses only 13 features established by Pafnucy; therefore, it does not use the 5 properties encoded by SMARTS patterns (hydrophobic, aromatic, acceptor, donor, and ring).

Bonds are summarized in an adjacency matrix having a size of NxN, with N being the number of nodes. In the adjacency matrix, the adjacent atoms are defined by a distance maximum of 4 Å for intermolecular distances and 2 Å for intramolecular distances. It is possible to train the neural network with up to 8 adjacency matrices. If the number of adjacency matrices is increased, the distance range covered by each is reduced. For example, in the case of using only one matrix, this one would cover interactions up to 4 Å. While in the case of using two adjacency matrices, the first one would account for interactions up to 2 Å, and the other one deals with interactions from 2 to 4 Å. The model established with two matrices achieved the best performance.

For data augmentation purposes, docking was performed, and the best poses with less than 3 Å of RMSD were selected—up to 3 poses. 

GraphBAR was trained on the PDBbind 2016 while discarding the complexes (pocket + ligand) containing too many atoms (>200 atoms). The models achieved coefficient correlations of 0.76 on the core set 2016 and 0.70 on the core set 2013. The data augmentation provided little improvements on the core set 2016 with a coefficient correlation of 0.78, and no improvements were measured for the core set 2013.

Performance was replicated using the code available here: https://github.com/jtson82/graphbar (accessed on 8 November 2023).

We carried out each experiment by replicating the training 10 times. All model replicates were performed in the same conditions, i.e., with the same neural network, the same hyper-parameters, and the same input data but different weights (randomized seeds) at the initialization of the neural network. The results were averaged, and the standard deviation was calculated in order to compare the performance of each experiment.

Models were trained with our laboratory cluster on graphics processing units (RTX 2080 and RTX 3090).

### 3.3. Metrics

The model performance was evaluated by predicting the binding affinity of each complex of test sets and comparing the results with real values. Prediction error was measured with the root mean square error (RMSE).

(1) $$RMSE=\sqrt{\frac{\sum_{i=1}^{N}{\left(x_{i}-{\hat{x}}_{i}\right)}^{2}}{N}}$$

The correlation between predicted binding affinity and the experimentally measured binding affinity was assessed using the Pearson correlation coefficient (R) and its standard deviation (SD).

(2) $$R=\frac{\sum \left(x_{i}-\bar{x}\right)\left(y_{i}-\bar{y}\right)}{\sqrt{\sum {\left(x_{i}-\bar{x}\right)}^{2}\Sigma {\left(y_{i}-\bar{y}\right)}^{2}}}$$

Statistical plots were performed with the library statannot (https://pypi.org/project/statannot/ (accessed on 8 November 2023)). Assuming normal distribution, all comparisons were performed with independent sample Student *t*-test with Bonferroni correction. The following p-values correspond to the annotations on the plots:

ns: 5.00 × 10^−2^ < *p* ≤ 1.00 × 10^0^
*: 1.00 × 10^−2^ < *p* ≤ 5.00 × 10^−2^
**: 1.00 × 10^−3^ < *p* ≤ 1.00 × 10^−2^
***: 1.00 × 10^−4^ < *p* ≤ 1.00 × 10^−3^
****: *p* ≤ 1.00 × 10^−4^

## 4. Conclusions

For some years now, deep learning models have been developed to predict protein–ligand binding affinity using structural data. The scientific community has been trying to establish guidance on how to use these tools. Data play a central role in training DL models. Therefore, we have been investigating how the data can impact the performance of models, as well as the intrinsic biases from the PDBbind. Among all the problems related to the data, the question of the quality and quantity of the data used to train DL algorithms seems crucial. For instance, another study has delved into the influence of the quantity and quality of nonstructural data on predicting binding affinities using deep learning [87]. Additionally, in structure-based affinity prediction, a lot of neural networks have been trained only on the PDBbind’s refined set instead of the totality of the data available. The refined set is made of complexes selected based on quality criteria. The reasoning for training on only the refined set is to avoid the “garbage in, garbage out” issue. We have evaluated this factor by training Pafnucy, a well-known CNN for the prediction of protein–ligand binding affinity, on the refined set only and on the entire dataset. We found that the performance was lower by training on the refined set. Therefore, we think it is important to train on most of the data available, as long as the data have been accurately labelled.

The PDBbind database groups several types of ligands together, with peptides and small molecules being the main populations involved in protein–ligand complexes. As only a few neural networks [15] have focused on training on complexes involving a specific type of ligand, we trained Pafnucy on the protein–peptide and protein–nonpeptide complexes of the PDBbind. We compared the performance by training on similar-sized datasets and found that models trained with peptides were able to better predict the binding affinity of protein–peptide complexes. Therefore, it would be interesting to investigate transfer learning on such type of data to reach good performance for the prediction of binding affinity of protein–peptide complexes.

Due to the computationally expensive nature of CNNs and their high requirement for RAM, it is not possible to train models on the whole protein structure. Indeed, beforehand, it is required to create pockets around the ligands. We have evaluated the performance of models trained on pockets made of the amino acids detected at 6, 8, 10, 12, and 14 Å from the ligand. By increasing the size of the pockets, we see performance increase until 10 Å; thereafter, performance stagnates. This performance trend, increasing with pocket size until reaching a certain value, aligns with OnioNet 2 [47], which showed performance improvement up to 15 Å. As most protein–ligand interactions should be already considered at a distance of 6 Å from the ligand, we propose that the increase in performance is due to the bias in the data. In other words, adding more information about the proteins would not add any useful physical information but just help the models to overfit. Another possible explanation would be related to the existence of some long-distance influences of these amino acids on the ligand, which would impact the affinity of the complexes. Therefore, the AI would detect these indirect interactions that would be hard to notice for a human.

Following on the topic of biases in the PDBbind core set, we evaluated different types of neural networks for their ability to learn from the interactions instead of memorizing the biases in the data. From these results, it seems that GraphBar does mostly QSAR since it has nearly the same performance with and without the proteins, or, in other words, Pafnucy seems to better understand the interaction between the protein and the ligand. On that topic, published work [13,19] reported even bigger performance gaps.

Finally, we pointed out some flaws inside the PDBbind 2016 core set. For example, 30% of the ligands from the test set are also in the PDBbind general set. As for the proteins, this value goes up to 100%. In the GNINA CNN v2017 publication [40], this was mitigated by removing test targets with more than 80% sequence similarity with a target from the training set. In a similar fashion, PIGNet [37] excluded from the CSAR NRC-HiQ the complexes that have at least 60% sequence similarity with a target from the training set. Following these examples, Yang et al. [17] advocate for the removal from test sets of complexes with structurally similar proteins and ligands in comparison to training sets. However, doing this prevents the evaluation of models in the situation of drug repurposing and hit-to-lead optimization [7]. Therefore, we recommend evaluating models on several test sets to better assess their ability to generalize and to accurately predict the binding affinity. On top of the CASF and the CSAR NRC-HIQ, we can list the Astex diverse set, the FEP dataset, and the holdout test sets. Several neural networks have already evaluated their performance on such datasets, allowing for easier comparison with the newly developed methodologies.

For a thorough evaluation of the models, we also advise evaluating their screening power. To measure that criterion, it is required to dock active molecules and decoys before evaluating their binding affinities and ranking the molecules. Some datasets propose a list of decoys and active molecules, like the DUD-E [66], DEKOIS [70], MUV [88], or the “Riniker and Landrum CHEMBL” [75]. The difference between these datasets depends mostly on the way they defined decoys and how they tried to prevent the appearance of biases. Unfortunately, biases can still be found in these datasets. In the end, models trained on the PDBbind did not outperform docking software in terms of vs. power when applied on the DUD-E [67]. Nonetheless, if it is possible to obtain better vs. power, even at the expense of lowering scoring power performance on the PDBbind core set, this would mean we are likely going in the right direction. This should be achievable by training models on a decoy pose-augmented PDBbind dataset, which should force models to learn from the interactions instead of memorizing ligand and protein structures. However, by using decoy poses, we might not accurately represent the physicochemical reality of the interactions of a protein and a ligand. Indeed, the interactions between them are dynamic; thus, the ligand might take several positions inside the binding site across time. As mentioned previously in the literature [89], it would be more suitable to perform data augmentation with molecular dynamics simulations. For example, snapshots could be extracted from the simulations and fed to neural networks. This way, we can expect to improve a model’s understanding of protein–ligand interactions.

## Figures and Tables

**Table 1:** Replication of results from three neural networks (Pafnucy, GraphBAR, and OctSurf) compared to the results presented in their respective publications. Models are evaluated based on their correlation coefficients and RMSE on the PDBbind core set 2016 (test set of 285 complexes).

Neural Network	Results from Publication		Results from Replication	
Pafnucy	R = 0.78 ^1^	RMSE = 1.42 ^1^	R = 0.77 SD = 0.01 ^1^	RMSE = 1.41 SD = 0.01 ^1^
GraphBAR	R = 0.76 ^1^	RMSE = 1.44 ^1^	R = 0.76 SD = 0.02 ^3^	RMSE = 1.43 SD = 0.03 ^3^
OctSurf	R = 0.79 ± 0.01 ^2^	RMSE = 1.45 ± 0.02 ^2^	R = 0.79 SD = 0.01 ^2^	RMSE = 1.46 SD = 0.03 ^2^

Training on: ^1^ PDBbind v2016 (13,308 complexes); ^2^ PDBbind v2018 (16,151 complexes); ^3^ PDBbind v2019 (17,679 complexes).

**Table 2:** Comparison of performance of several neural networks on the PDBbind core set 2016 (test set of 285 complexes) with/without protein/ligand.

Neural Network	Whole Complex (R, RMSE)	Only Ligand (R, RMSE)	Only Protein (R, RMSE)
Pafnucy ^1^	0.77, 1.41	0.65, 1.67	0.66, 1.64
GraphBAR ^2^	0.76, 1.43	0.73, 1.51	0.59, 1.77
OctSurf ^1^	0.79, 1.45	0.73 (n.a.)	0.64 (n.a.)
Modular MPNN ^2^ [7]	0.81, 1.51	0.75, 1.57	0.73, 1.57
Deep Fusion ^3^ [13]	0.81, 1.31	0.49, 3.01	0.5, 4.00
PointTransformer ^4^ [19]	0.86, 1.19	0.45 (n.a.)	0.2 (n.a.)

Types of neural networks: ^1^ 3D convolutional neural network (3D-CNN); ^2^ Graph neural network (GNN); ^3^ 3D-CNN + spatial graph CNN (SG-CNN); ^4^ Transformer; n.a., not available.

## Data Availability

The data presented in this study are openly available in the PDBbind at 10.1021/jm048957q.

## Supplementary Materials

The supporting information can be downloaded at: https://www.mdpi.com/article/10.3390/ijms242216120/s1.

## Appendix A

**Table A1:** Number of complexes of the CSAR NRC-HiQ set 1 & 2, used in each publication. In GIGN, the sets were merged together.

Neural Network	CSAR NRC-HiQ set1	CSAR NRC-HiQ set2
K_DEEP_ [46]	55	49
RosENet [44]	33	10
OnionNet-2 [47]	55	49
graphDelta [48]	53	49
GraphBAR [35]	51	36
PIGNet [37]	48 & 37	37 & 22
BAPA [49]	50	44
CAPLA [50]	51	36
GIGN [21]	47	

**Table A2:** Summary of the FEP dataset from K_DEEP_ [46] and Wang et al. [27]. This table displays the target (protein family), the reference PDB id used, the number of ligands positioned in 3D in each structure, and the experimental affinity range of complexes belonging to the same protein family.

Target	PDB ID	Number of Ligands	Affinity Range (kcal/mol)
MCL1	4HW3	42	4.2
BACE	4DJW	36	3.5
p38	3FLY	34	3.8
PTP1B	2QBS	23	5.1
JNK1	2GMX	21	3.4
CDK2	1H1Q	16	4.2
Tyk2	4GIH	16	4.3
Thrombin	2ZFF	11	1.7
